# Editorial: Active Aging and Disease Management

**DOI:** 10.3389/fmed.2018.00278

**Published:** 2018-10-05

**Authors:** Helena Canhao, Jaime C. Branco, Giuseppe Liotta

**Affiliations:** ^1^CEDOC, EpiDoC Unit, NOVA Medical School, Universidade NOVA de Lisboa, Lisbon, Portugal; ^2^CHLO - Hospital Egas Moniz, EPE, Lisbon, Portugal; ^3^Biomedicine and Prevention Department, Università degli Studi di Roma Tor Vergata, Rome, Italy

**Keywords:** aging, health, disease management, active, research topic

With population aging and an increasing number of older people in countries worldwide, issues such as the advancement of healthy and active aging of the individual as well as the sustainability of health care systems are of growing importance. Multimorbidity is more prevalent with advancing age, as are other factors, such as social isolation and cognitive and physical impairment. These factors result in increased mortality and a greater use of health care services by older people. Polypharmacy is a well-documented risk factor for poor health outcomes in older people, and this is particularly true for those of low income and literacy. Adequate nutrition is essential for promoting active aging, and malnutrition is a key element in many disease processes at advanced age.

All these challenging issues justify the edition of our Research Topic “Active Ageing and Disease Management” which, as we expect, can bring up some of the problems and point out good practices to tackle these health and social needs. This group of papers has in common a holistic perspective focused on health promotion and disease prevention.

This research topic gathers 14 papers from different research groups that deal with distinct aspects of active aging and disease management. Three of them are opinion articles, nine original research, one clinical trial, and one community case study.

Liotta et al. in “Active Ageing in Europe: Adding Healthy Life to Years,” emphasize the key role of the determinants of health in active and healthy aging. They recall that, in the nineteenth century, an improvement of socioeconomic conditions, environmental sanitation and nutrition started a public health revolution that ultimately allowed an increase of life expectancy from an average of 45 years old to the average of ~80 years old that we observe now at almost European countries. Strengthening the social dimension of prevention programmes and changing the lifestyles are key elements to improve quality of life and reduce the risk of loss of physical autonomy, especially among the over-75 population. Real social and health care integration at community level could be the key point to deliver effective health promotion and preventive intervention.

O'Caoimh et al. in the paper “Can the Geriatric Day Hospital Act As a Hub for Services for Older People across the Spectrum of Ageing from Active Ageing to Advanced Frailty?” discussed the potential role to be played by the Geriatric Day Hospital as a hub to manage the care of older adults with complex needs across the spectrum from active aging to pre-frailty and from established frailty to end-of-life care. To accomplish that, Day Hospitals should focus on providing innovative and proactive, preventive approaches including those that use new mobile ICT technologies to promote healthy aging, address pre-frailty and prevent or reverse frailty at an early stage, before the onset of functional decline.

Kyriazis and Kiourti in the opinion paper “Video Games and Other Online Activities May Improve Health in Ageing,” argued that ICTs stimulus might act as a cognitive challenge that upregulates neuronal stress response pathways, the same way as other challenges and stressors that result in “positive stress.” This upregulates neuronal health in older people and their use should be encouraged.

Original research brought up different hot topics, stressing the role played by nutrition, physical exercise, and healthy lifestyles in older people.

Fernandes et al. reported on “Food Insecurity in Older Adults: Results From the Epidemiology of Chronic Diseases Cohort Study 3 that food insecurity is associated with higher likelihood for chronic diseases, poor self-management, and lower HRQoL and should be fought with adequate policies. Tomás et al. with “Functional Capacity and Levels of Physical Activity in Ageing: A 3-Year Follow-up,” Dekker-van Weering et al. with “User Experience, Actual Use, and Effectiveness of an Information Communication Technology-Supported Home Exercise Program for Pre-Frail Older Adults” and Oliveira et al. with “Kinematic Changes during Prolonged Fast-Walking in Old and Young Adults” highlighted the benefits of physical exercise, namely walking and activities that use hands, since these specific components seem to have a major impact in functional physical condition. The second study provides evidence that a home-based exercise program is easy to use and has potential on improving quality of life and health status of physically pre-frail, older adults who live at home. The last one showed that fast walking induces changes over time on kinematics of old adults, mainly at ankle and hip, and in the coordination among the lower-limb angles that were more prominent during the swing phase of the gait and may indicate an augmented risk of falling. These findings provide a foundation for future studies in the assessment of the risk of falls in older adults associated with walking at a faster pace.

Another interesting paper from Duarte et al. “Health and Lifestyles Factors Associated With Osteoarthritis among Older Adults in Portugal,” showed the independent association of osteoarthritis with age, female gender, higher number of comorbidities, physical disability, and low levels of physical activity.

When thinking of old people taking into account their multidimensional aspects, psychological and cognitive issues have a key role. Three papers address these questions: “Active Ageing in Very Old Age and the Relevance of Psychological Aspects” from Paúl et al. “Anxiety and Depression in the Portuguese Older Adults: Prevalence and Associated Factors” by Sousa et al. and “Neuropsychological Correlates of Pre-Frailty in Neurocognitive Disorders: A Possible Role for Metacognitive Dysfunction and Mood Changes” from Amanzio et al. Psychological aspects proved to be of great relevance for active aging, and corroborate previous research that consider mental health balance as an important contributor to an optimist view of life and cognitive capacity. Prevalence of anxiety and depression among elders is around 10–12%, and health-related quality of life and physical function play an important role in depression and anxiety. In patients with neurocognitive disorders such as Alzheimer's disease, cognitive functions can have a significant role in the pathogenic mechanisms of frailty, establishing a link between them.

The last three papers deal with three distinct but very important subjects. In “Ageing, Disability, and Informal Caregivers: A Cross-sectional Study in Portugal,” Pego and Nunes stressed the key role of informal caregivers. A significant proportion of dependent older people lacks any kind of informal care (39.5%), which supports the argument that the current limitations of the long-term care model impose a revolution of the community care model to be addressed. The paper underlines the marked nuclearization of the family and solidarity networks mainly composed of family members. Geographical distance also is extremely relevant to the frequency of care, i.e., the closer the caregiver lives, the higher the frequency of care provided.

The results of this study show a critical situation for both groups (elderly and caregivers) and an alarming prospect in the near future due to the reduced availability of informal young caregivers to care for the older person; this is a global challenge, since many countries share the same characteristics. The community care model cannot ignore these challenges; otherwise, they drive to fail the whole care systems due to the potential increase of demand in the next years.

Gonçalves et al. developed a new tool, the “Selfie Ageing Index: An Index for the Self-assessment of Healthy and Active Ageing.” SAI measures healthy and active aging at the individual level and was designed for individuals to monitor their aging status. For this reason, it is completely based on self-assessed indicators, without requiring a health-care professional to complete it—a new approach to the community care that is particularly needed to screen large population. The SAI has the potential to put the individual at the center of the aging discussion, contributing to patient empowerment and promoting patient-centered care.

Finally, in “The Quadruple Helix-Based Innovation Model of Reference Sites for Active and Healthy Ageing in Europe: The Ageing@Coimbra Case Study,” Malva et al. reported on the innovative and successful example of Ageing@Coimbra as a Reference Site of European Innovation Partnership on Active and Healthy Ageing. It supports the adoption of bottom-up, inclusive, and holistic approaches to create regional networks and partnerships with the aim of supporting the creation and replication of good practices for Active and Healthy Ageing. It is a good example of a forum to elaborate shared policy for the older adults' QoL, which will be spread at European level.

We believe the papers show a comprehensive vision of relevant aspects of active aging and disease management and help to increase knowledge and awareness around these themes. Enjoy the reading!

## Author contributions

All authors listed have made a substantial, direct and intellectual contribution to the work, and approved it for publication.

### Conflict of interest statement

The authors declare that the research was conducted in the absence of any commercial or financial relationships that could be construed as a potential conflict of interest.

